# Detailed analysis of the histology-specific impact of ascites volume on the outcome of epithelial ovarian cancer: a multi-institutional retrospective cohort study

**DOI:** 10.1186/s12885-024-13218-1

**Published:** 2024-11-29

**Authors:** Shohei Iyoshi, Mariko Kimura, Masato Yoshihara, Atsushi Kunishima, Emiri Miyamoto, Hiroki Fujimoto, Kazuhisa Kitami, Kazumasa Mogi, Kaname Uno, Sho Tano, Nobuhisa Yoshikawa, Ryo Emoto, Shigeyuki Matsui, Hiroaki Kajiyama

**Affiliations:** 1 Department of Obstetrics and Gynecology, Nagoya University Graduate School of Medicine, 65 Tsurumai-cho, Showa-ku, Nagoya, 466-8550 Japan; 2 Institute for Advanced Research, Nagoya University, Furo-cho, Chikusa-ku, Nagoya, 464-8601 Japan; 3 Department of Obstetrics and Gynecology, Okazaki City Hospital, 3-1 Goshoai, Kouryuji-cho, Okazaki, 444-8553 Japan; 4 Discipline of Obstetrics and Gynaecology, Adelaide Medical School, Robinson Research Institute, University of Adelaide, Adelaide, SA Australia; 5 Department of Gynecologic Oncology, Aichi Cancer Center, 1-1 Kanokoden, Chikusa-ku, Nagoya, 464-8681 Japan; 6 Department of Biostatistics, Nagoya University Graduate School of Medicine, Nagoya, Japan

**Keywords:** Ovarian cancer, Ascites volume, Multi-institutional study

## Abstract

**Background:**

The accumulation of ascites is a major symptom of ovarian cancer. The volume of ascites is a pathophysiological indicator of the peritoneal environment, such as inflammation and fibrosis; however, the relationship between the volume of ascites and oncological outcomes remains unclear. We herein retrospectively examined the effects of the volume of ascites on the prognosis of epithelial ovarian cancer in a multi-institutional large cohort using the stratification of clinical characteristics and statistical adjustment methods.

**Methods:**

Of 5,268 patients with ovarian tumors in the Tokai Ovarian Tumor Study Group between 1986 and 2020, we included 1,966 cases of epithelial ovarian cancer and examined the relationship between the volume of ascites at the initial surgery and the prognosis of patients. We performed a multivariate analysis and propensity score weighting for covariate adjustments to precisely estimate the prognostic impact of ascites accumulation. A subgroup analysis was also performed to examine differences in the prognostic implications of ascites accumulation among histotypes.

**Results:**

A reservoir of 100 mL of ascites was confirmed as the cut-off value in our cohort. A Kaplan-Meyer analysis with propensity score adjustments indicated that the accumulation of more than 100 mL of ascites shortened overall survival. The multivariate analysis revealed that the increased accumulation of 100 mL of ascites was an independent prognostic factor for overall survival (HR 1.242; 95% CI 1.050–1.470; *P* = 0.012). The subgroup analysis showed the prognostic significance of ascites accumulation in mucinous and endometrioid histologies.

**Conclusions:**

The accumulation of even a low to intermediate volume of ascites (≥ 100 mL) was confirmed to be an independent poor prognostic factor in epithelial ovarian cancer. Furthermore, its prognostic impact differed among histotypes.

**Supplementary Information:**

The online version contains supplementary material available at 10.1186/s12885-024-13218-1.

## Background

Ovarian cancer (OvCa) does not cause specific symptoms in its early stage and, thus, most cases are diagnosed at an advanced stage [1]. At the time of diagnosis, the majority of patients have fluid accumulation in the peritoneal cavity, which is called ascites [2]. It has long been assumed that OvCa cells dispatched from primary lesions utilize the accumulation of ascites to float as a form of spheroid and then directly metastasize into the peritoneum, leading to multiple disseminated nodules within the peritoneal cavity [3–5]. Accumulated ascites reflects the status or condition of the whole peritoneal ecosystem, including inflammation, fibrosis, vascular permeability, and the absorption capacity of the peritoneum [6–8]. Therefore, the accumulation of ascites is of fundamental significance in the pathophysiology and progression of OvCa [9, 10].

Previous studies highlighted the prognostic significance of the volume of ascites. Nasioudis et al. examined a national cancer database and revealed that patients with a large volume of ascites (more than 980 mL) at primary debulking surgery (PDS) had a lower rate of complete gross resection and shorter overall survival (OS) [11, 12]. Single or dual institutional studies also concluded that patients with a high volume of ascites at PDS had a poorer prognosis; shorter progression-free survival (PFS) and OS, and an increased risk of disease recurrence [13–16]. Janco et al. utilized computed tomography (CT) to estimate the volume of ascites in a single-center retrospective cohort study and concluded that the absence of ascites was an independent predictor of complete cytoreduction in debulking surgery [15]. Therefore, the volume of ascites has a prognostic impact in OvCa. However, since the majority of studies focused on the significance of massive ascites accumulation (more than ca. 1000 mL), the prognostic impact of a low to intermediate volume (100–1000 mL) of ascites remains unclear. Moreover, most of these studies, except those using national cancer databases, were conducted at one or two institutions and, thus, detailed analyses, for example, differences in the prognostic impact of the volume of ascites among histotypes, were not conducted because of the limited number of cases examined.

Therefore, we herein investigated the comprehensive impact of the volume of ascites on the prognosis of epithelial OvCa in a multi-institutional large-scale clinical cohort. To accurately estimate prognostic significance, adjustments by the propensity score (PS) weighting method were performed. Detailed subgroup analyses, including stratification by clinical stages and histotypes, were also conducted.

## Methods

### Study participants

We conducted a multi-institutional retrospective cohort study on data of the Tokai Ovarian Tumor Study Group, comprising Nagoya University Hospital and affiliated institutions. This study was approved by the Ethics Committee of Nagoya University (No. 2006 − 0357) in accordance with the principles of the Declaration of Helsinki. Data were accumulated from the medical records of each institution between 1986 and 2020. Written informed consent was waived for some participants because the present study did not include any individual information. Histopathological slides were reviewed by expert central pathologists according to the criteria of the World Health Organization classification [17] and patients diagnosed with epithelial OvCa were included in the present study. Patients lost to the follow-up after the initial surgery or those without sufficient clinical information were excluded. Clinical staging was performed using the FIGO surgical staging system [18] and the tumor-node-metastasis (TNM) pathological classification [19] based on sufficient clinical data.

### Surgery, chemotherapy, and follow-up

PDS or interval debulking surgery was performed on all patients. The procedure consisted of complete-staging surgery, including total hysterectomy and bilateral salpingo-oophorectomy with a full peritoneal evaluation plus ascites cytology, biopsy, and/or omentectomy, lymphadenectomy, and peritoneal exploration [20, 21]. We defined complete-staging lymphadenectomy as resection of the pelvic and para-aortic lymph nodes. Some patients underwent incomplete surgery, including uterine preservation and the omission of staging lymphadenectomy for clinical reasons, such as the sparing of fertility. All patients received chemotherapy, the contents of which in each period were described in our previous study [22]. Patients were followed up with evaluations of tumor markers and regular pelvic examinations using ultrasonography, magnetic resonance imaging, computed tomography, or positron emission tomography. Recurrence was diagnosed as the development of ascites, a detectable mass, or elevated tumor markers according to the criteria of the Gynecologic Cancer InterGroup [23]. PFS and OS were defined as the time from the date of initial surgery to that of the last follow-up or tumor recurrence and death, respectively.

### Statistical analysis

Statistical analyses were performed using R statistical software (version 4.2.3) and Rstudio (version 2023.06.0 + 421). A PS-based method was used to adjust imbalances between the ascites high (≥ 100 mL) and low (< 100 mL) groups, with scores being calculated using a logistic regression model of the original population [24]. Study cohorts were adjusted by inverse probability weighting of the treatment approach, in which each individual was weighted by the inverse probability of low and high volumes of ascites [25]. Comparisons of continuous variables between groups were performed using the Student’s *t*-test (normal distribution) and Mann-Whitney U test (non-normal distribution). The chi-squared or Fisher’s exact test were used for categorical variables. When analyzing the prognosis of patients, the Kaplan–Meier method was used to compare peritoneum-specific recurrence-free survival, recurrence-free survival, and OS.

Differences in survival between two groups were assessed by the Log-rank test and Cox’s regression model. In the subgroup analysis, the adjusted estimation of the hazard ratio (HR) of ascites accumulation (≥ 100 mL) was also performed by stratifying each variable, and conducting multivariate Cox’s regression (Variables; Age, FIGO staging, logged CA-125, Ascites cytology, and adjuvant chemotherapy. The variable used in stratification was dropped). Significance was selected as two-sided with a P value < 0.05.

## Results

### Baseline characteristics of patients

**Fig. 1:**
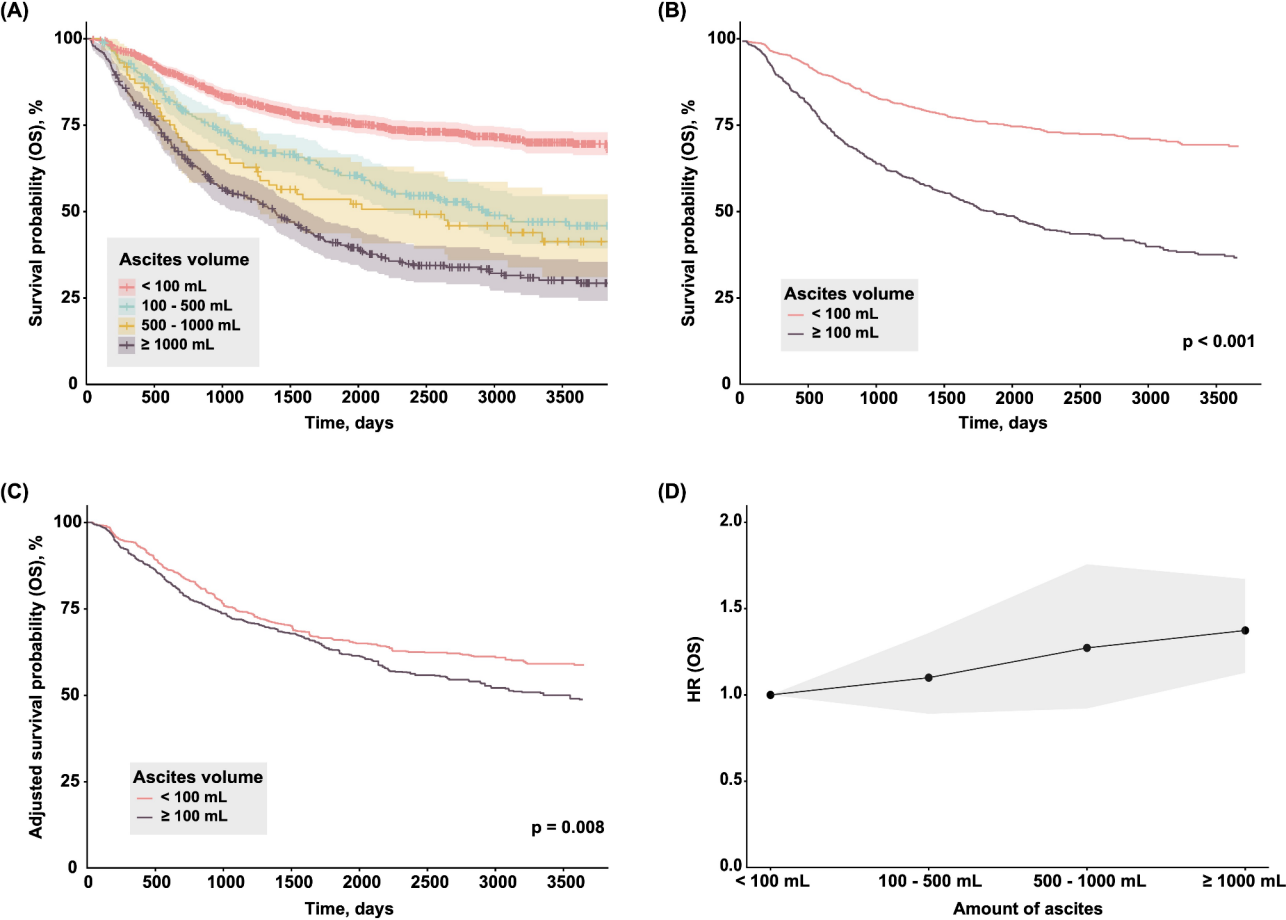
(**A**) Kaplan–Meier curves for OS stratified by four ascites volume classes (≤ 100 mL, 100–500 mL, 500–1000 mL, and ≥ 1000 mL). (**B**-**C**) Kaplan–Meier curves for OS stratified by two ascites classes (< 100 mL and ≥ 100 mL) with (**B**) unadjusted and (**C**) adjusted cohorts. (**D**) HR values for OS in each ascites volume class (the HR value of the lowest ascites volume < 100 mL was used as a reference) were plotted and 95% confidence intervals were shown as a gray belt

Among 5,268 patients with ovarian tumors, 1,966 were selected according to the study criteria (Figure S1). Based on the volume of ascites measured at the initial cytoreductive surgery, patients were classified into the following four groups: ≤100 mL (*n* = 1,189), 100–500 mL (*n* = 319), 500–1000 mL (*n* = 86), and ≥ 1000 mL (*n* = 372). When Kaplan-Meier curves among these four groups were drawn, a significant difference was observed between the first group and the latter 3 groups (Fig. 1A). Based on this result, we set an ascites volume of 100 mL as the threshold and divided our cohort into two groups. Among 1,966 cases, 777 and 1,189 were classified into the low ascites volume group (< 100 mL) and high ascites volume group (≥ 100 mL), respectively. The baseline characteristics of these two groups are summarized in Table 1. As expected, clinical staging affected the accumulation of ascites and a high volume of ascites was more common in advanced stages (stage III/IV). The cytology of ascites was more likely to become negative and R0 resection, a surgical procedures that result in no visible residual disease [26, 27], was more easily achieved in the ascites low group. Furthermore, from a histological aspect, the volume of ascites was slightly higher with a serous histology and lower with a endometriosis-related histology, including clear-cell and endometrioid carcinoma (Table S1).

**Table 1:** Baseline characteristics of patient in low and high ascites volume classes

	Ascites volume		*p*-value
	High (< 100 mL)	Low (≥ 100 mL)	
n	777	1189	
Age, years (SD)	55.35 (11.45)	54.99 (11.90)	0.514
Tumor stage, n (%)			
I	186 (23.9)	661 (55.6)	< 0.001
II	91 (11.7)	157 (13.2)	0.365
III	416 (53.5)	300 (25.2)	< 0.001
IV	84 (10.8)	71 ( 6.0)	< 0.001
Hisototype, n (%)			
Serous	372 (47.9)	337 (28.3)	< 0.001
Clear-cell	171 (22.0)	419 (35.2)	< 0.001
Mucinous	88 (11.3)	140 (11.8)	0.817
Endometrioid	118 (15.2)	260 (21.9)	< 0.001
Others (%)	28 ( 3.6)	33 ( 2.8)	0.367
CA125, IU/mL (SD)	1888.28 (4021.66)	914.99 (3765.41)	*<0.001
Resection, n (%)			
R0	342 (44.0)	973 (81.8)	< 0.001
R1	435 (56.0)	216 (18.2)	< 0.001
Ascites cytology, n (%)			
Positive	546 (70.3)	384 (32.3)	< 0.001
Negative	231 (29.7)	805 (67.7)	< 0.001
Chemotherapy, n (%)	734 (94.5)	1003 (84.4)	< 0.001

Data are presented as mean ± standard deviation or proportion (%)

Student’s *t*-test, chi-square test, or Fisher’s exact test was used as appropriate

Abbreviations: SD, standard deviation; CA, cancer antigen

* Logarithmically transformed when analyzed

### Impact of ascites volume in epithelial OvCa

The effects of clinical factors, including ascites volume, were estimated as HR for PFS and OS in all patients (Table 2). In the multivariate analysis, the volume of ascites correlated with poor OS [HR, 1.242, 95% confidence interval (CI) 1.050–1.470, *p* < 0.012], but not PFS [HR, 1.097, 95% CI 0.932–1.290, *p* = 0.267]. Kaplan-Meier curves of the low and high ascites groups are shown in Fig. 1B, and indicated the markedly different clinical outcomes of each ascites group for OS. We then assessed the prognostic impact of ascites volume with PS adjustments on the following five clinical factors that have been shown to affect the survival outcomes of patients: age, stage (I/II vs. III/IV), histology (serous vs. others), CA-125 levels, and the performance of complete staging surgery. Kaplan-Meier curves with PS-based IPTW adjustments revealed that a high volume of ascites was associated with a significantly worse prognosis (Fig. 1C). Moreover, HR was more likely to be higher as the volume of ascites increased (Fig. 1D). Similar results were obtained for PFS, namely, shorter survival in patients with a higher volume of ascites; however, the difference was not significant after PS-based adjustments when 100 mL of ascites was used as the cut-off value (Figure S2 A-D).

**Table 2:** Uni and multivariate analysis

	PFS					OS				
	Univariate			Multivariate		Univariate			Multivariate	
	HR (95% CI)	*p*-value		HR (95% CI)	*p*-value	HR (95% CI)	*p*-value		HR (95% CI)	*p*-value
FIGO stage										
I	ref	ref		ref	ref	ref	ref		ref	ref
II	1.69 (1.28–2.22)	< 0.001		1.32 (0.99–1.76)	0.061	2.53 (1.86–3.43)	< 0.001		2.12 (1.53–2.94)	< 0.001
III	5.49 (4.57–6.59)	< 0.001		3.07 (2.40–3.92)	< 0.001	7.43 (5.96–9.26)	< 0.001		4.21 (3.15–5.64)	< 0.001
IV	5.82 (4.47–7.60)	< 0.001		2.99 (2.16–4.13)	< 0.001	10.76 (8.19–14.13)	< 0.001		5.26 (3.74–7.40)	< 0.001
Histotype										
Serous	ref	ref		ref	ref	ref	ref		ref	ref
Clear	0.40 (0.34–0.48)	< 0.001		1.01 (0.82–1.25)	0.915	0.49 (0.41–0.59)	< 0.001		1.61 (1.30–1.98)	< 0.001
Mucinous	0.30 (0.22–0.40)	< 0.001		0.94 (0.68–1.30)	0.707	0.55 (0.42–0.70)	< 0.001		2.08 (1.56–2.77)	< 0.001
Endometrioid	0.32 (0.26–0.40)	< 0.001		0.76 (0.60–0.97)	0.028	0.31 (0.24–0.40)	< 0.001		0.89 (0.68–1.15)	< 0.366
Others	0.43 (0.27–0.69)	< 0.001		0.59 (0.37–0.95)	0.03	0.87 (0.59–1.28)	0.487		1.41 (0.95–2.08)	0.085
Age	1.02 (1.01–1.03)	< 0.001		1.01 (1.01–1.02)	< 0.001	1.02 (1.01–1.03)	< 0.001		1.01 (1.01–1.02)	< 0.001
CA125	1.29 (1.24–1.34)	< 0.001		1.05 (1.00-1.10)	0.052	1.28 (1.23–1.33)	< 0.001		1.00 (0.95–1.05)	0.934
R0/R1										
R1	ref	ref		ref	ref	ref	ref		ref	ref
R0	0.28 (0.24–0.32)	< 0.001		0.76 (0.63–0.91)	0.004	0.19 (0.16–0.22)	< 0.001		0.45 (0.37–0.55)	< 0.001
Ascites volume										
< 100 mL	ref	ref		ref	ref	ref	ref		ref	ref
≥ 100 mL	2.05 (1.78–2.37)	< 0.001		1.10 (0.93–1.29)	0.265	2.59 (2.23–3.01)	< 0.001		1.24 (1.05–1.47)	0.012
Ascite cytology										
Negative	ref	ref		ref	ref	ref	ref		ref	ref
Positive	3.03 (2.60–3.52)	< 0.001		1.45 (1.22–1.73)	< 0.001	3.89 (3.30–4.60)	< 0.001		1.66 (1.37–2.01)	< 0.001
Chemotherapy										
Adjuvant -	ref	ref		ref	ref	ref	ref		ref	ref
Adjuvant +	3.61 (2.51–5.20)	< 0.001		1.30 (0.87–1.95)	0.193	3.36 (2.28–4.93)	< 0.001		0.90 (0.58–1.38)	0.621

### Subgroup analysis

**Fig. 2:**
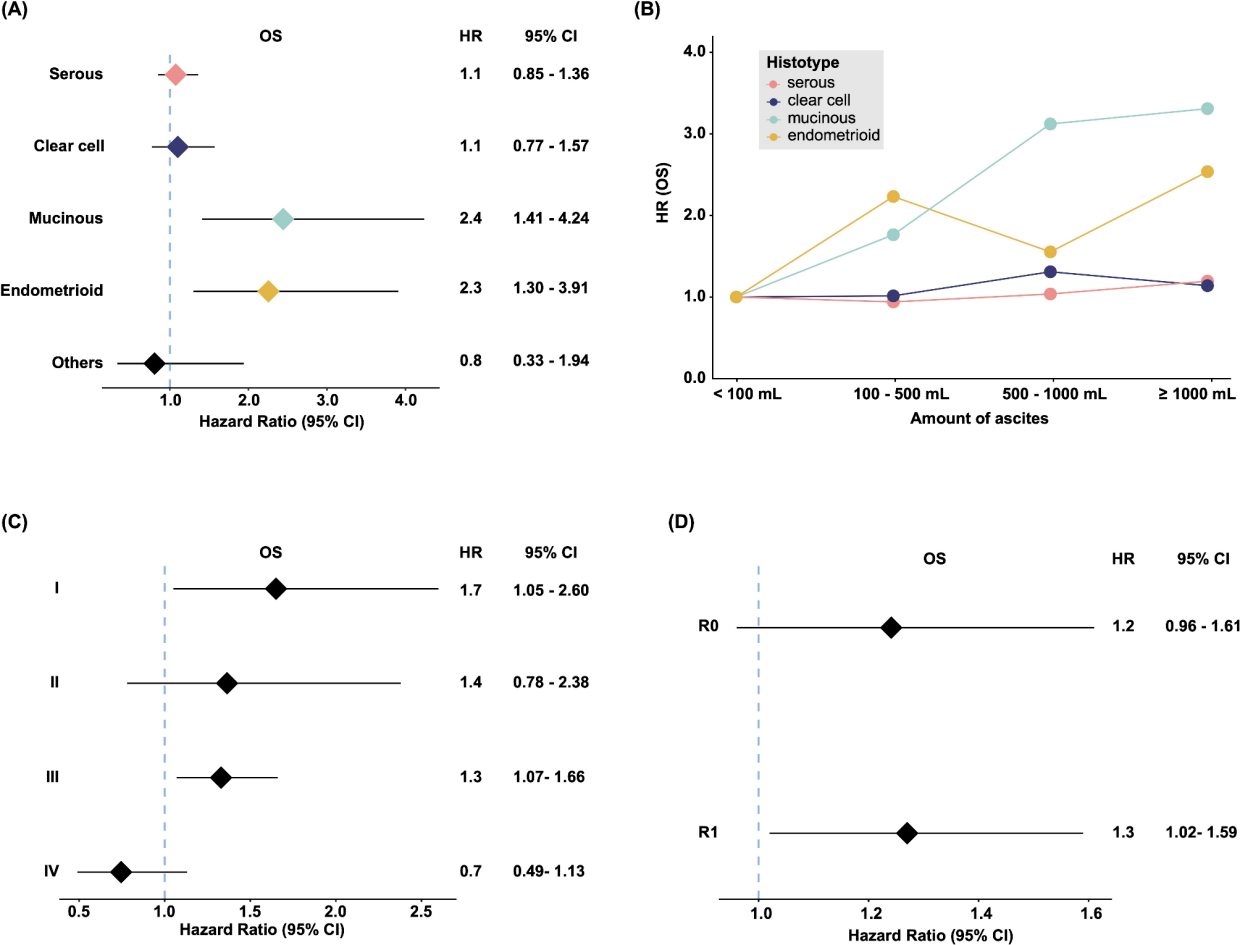
(**A**-**D**) Results of subgroup analyses of (**A**-**B**) histology, (**C**) FIGO staging, and (**D**) R0/R1 surgery groups depicted as HR values

**Fig. 3:**
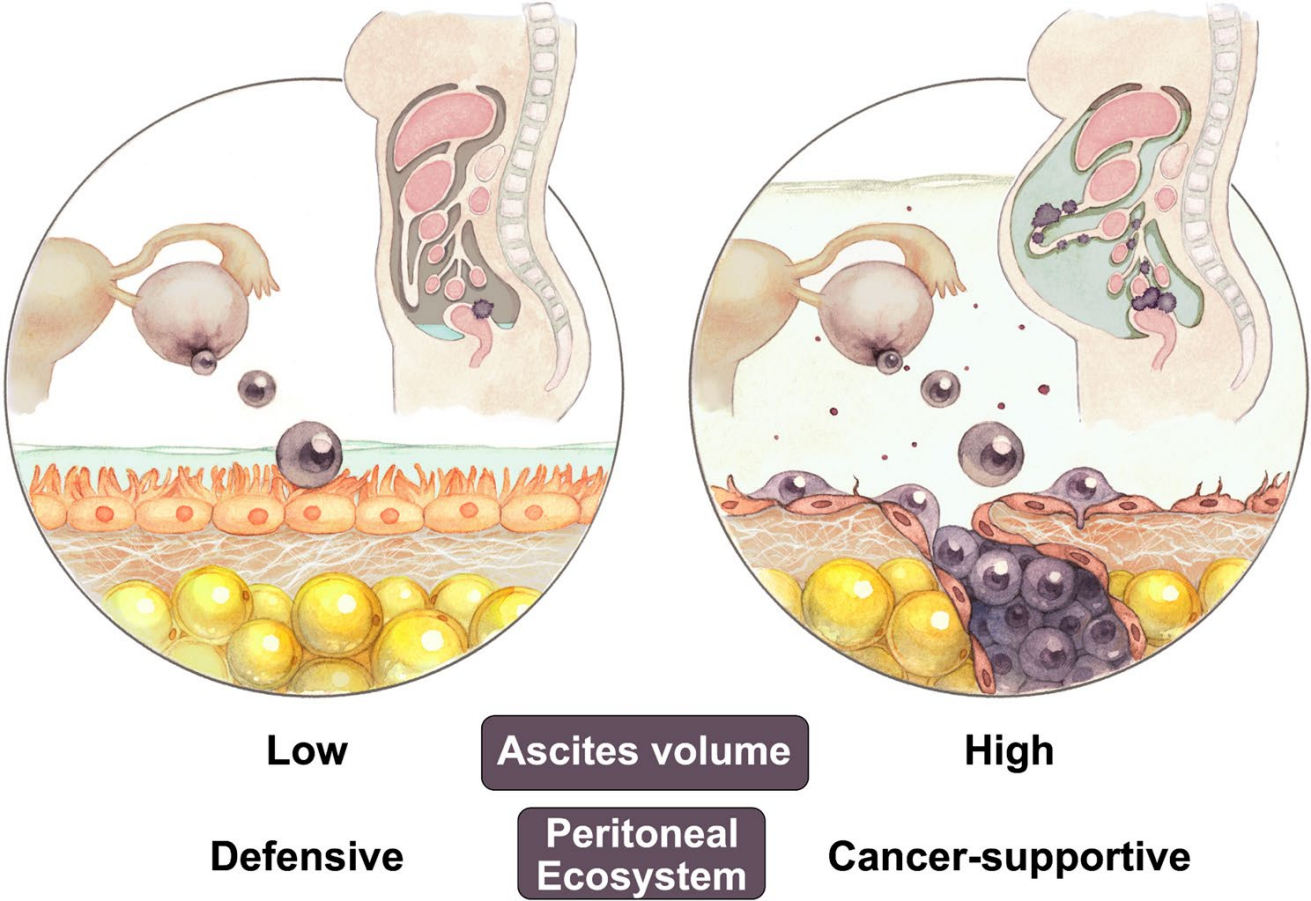
Schematic image based on the present results

The estimated relative HR values of death with a high volume of ascites for each clinical factor were then plotted (Fig. 2). We examined the effects of histotypes and confirmed that a high volume of ascites was more critical in cases of mucinous and endometrioid carcinoma (HR of OS, 2.44 and 2.26, *p* = 0.002 and 0.004, respectively). When the HR values of mucinous carcinoma cases were plotted against the volume of ascites, a gradual increase in HR was confirmed as the volume of ascites became larger (Fig. 2B). The effects of clinical staging indicated that the volume of ascites negatively affected prognosis in the stages to which most of the OvCa patients were classified, namely, FIGO Stages I and III (Fig. 2C). In addition, the effects of the volume of ascites on the prognosis of OvCa patients were not markedly affected by whether complete resection of the tumor in surgery was achieved (Fig. 2D). Similar results were obtained for PFS in subgroup analysis; however, the effect was slight. (Figure S3 A-D) (See Fig. 3).

## Discussion

The accumulation of ascites is a common symptom of OvCa and reflects the peritoneal environment, including inflammation and fibrosis [2, 5]. Since these peritoneal conditions are closely associated with oncological outcomes, the characteristics of ascites, including the volume at PDS, are of clinical significance. In the present study, with PS adjustments, even a low to intermediate volume of ascites (ascites volume ≥ 100 mL) was an independent prognostic factor in OvCa. The multivariate analysis showed that the retention of ascites significantly shortened OS (HR 1.202; 95%CI 1.014–1.425; *P* = 0.034), but not PFS. Furthermore, the subgroup analysis indicated that ascites volume ≥ 100 mL significantly shortened OS in stages I and III. The results obtained from our large-scale cohort revealed that the prognostic impact of ascites accumulation varied among histotypes; it was stronger for mucinous and endometrioid histotypes.

Previous studies reported the negative impact of a high volume of ascites on the prognosis of OvCa [11–16], which is consistent with the present results. However, most of these studies mainly investigated cases with a high volume of ascites > 1000 mL [11–14, 16] and, thus, the impact of a low to intermediate volume of ascites (≥ 100 mL) remains unclear. The present results showing the negative prognostic impact of even a low to intermediate volume (≥ 100 mL) of ascites has provided novel insights in the field. Furthermore, previous studies mainly focused on cases of the high-grade serous carcinoma histotype only. Based on a large-scale cohort from multiple institutions, the present study was the first to perform a subgroup analysis of histotypes and revealed variations in the prognostic impact of ascites accumulation among histotypes.

The volume of ascites is regulated by the dynamic equilibrium between capillary transudation or exudation and lymphatic absorption via the mesothelial peritoneum [2, 5]. The disruption of this equilibrium leads to the accumulation of ascites. In OvCa cases, ascites is typically exudative with high protein and cellular contents, and elevated protein levels in the fluid contribute to heightened osmotic pressure within the peritoneal cavity. Concurrently, various cytokines from cellular components induce peritoneal inflammation and damage to mesothelial cells impairs the drainage mechanism, resulting in the accumulation of ascites. Intraperitoneal physical stress and the resulting compression of adipose tissue are known to induce adipocyte dedifferentiation and promote tumor progression [28, 29]. Since adipose tissues, including the omentum and mesenteries, are the most frequent site of OvCa dissemination, compression induced by the massive accumulation of ascites may be one of the reasons why the volume of ascites is an independent prognostic factor. A low to intermediate volume of ascites does not induce significant compression in the peritoneal cavity and, thus, its negative impact on the prognosis of patients appears to involve a different mechanism. Ascites contains multiple signaling molecules and is considered to actively contribute to the creation of a specific tumor microenvironment for metastasizing OvCa cells. Supporting this, a previous study compared the effects of an acellular ascites or PBS injection into the peritoneal cavity of a syngeneic OvCa mouse model, and confirmed the greater negative impact of ascites [13]. Therefore, the accumulation of ascites is the driving force of tumor progression, but is not a marker of an advanced disease status.

The subgroup analysis of the histopathology of epithelial OvCa in the present study newly highlighted differences among histotypes. High-grade serous carcinoma is the most prevalent histotype and is known to create greater peritoneal dissemination, leading to damage in the peritoneum and the massive accumulation of ascites. Clear cell carcinoma is mainly diagnosed in the early stages; however, our cohort included a larger number of stage III cases than mucinous and endometrioid carcinoma. A low to intermediate volume of ascites did not have a prognostic impact in these two histotypes, which indicated that the volume of ascites merely reflected the disease status. Regarding mucinous and endometrioid carcinoma, the mild accumulation of ascites was of greater prognostic significance and became an independent prognostic factor. In addition, the accumulation of ascites was related to a poor prognosis in patients with stage III disease or a residual tumor in our cohort dataset. Since these conditions indicate the presence of abundant cancer cells in the peritoneal cavity as peritoneal dissemination, ascites appears to play an important role as a site for various interactions between cancer cells and the peritoneal environment, including post-translational modifications to secreted factors. Notably, the negative impact of a high ascites volume decreased as the disease stage increased. This would suggest that ascites accumulation in early-stage OvCa possess more prominent clinical significance than in advanced stage where symptoms including ascites appears more drastically and universally.

One of the strengths of this study is that we analyzed a large amount of cohort data with a central pathology diagnosis system, which minimized variance in pathological diagnoses. We also considered statistical processing using PS-IPTW to have contributed to the robustness of the results obtained. On the other hand, the number of cases in some subgroups was small and confounding factors may have existed due to the retrospective nature of the study. The future verification of these results using other validation cohorts is needed.

## Conclusion

The accumulation of ascites, even a low to intermediate volume (≥ 100 mL), was associated with a shorter OS, particularly in cases of mucinous and endometrioid carcinoma. The accumulation of ascites needs to be considered not merely as a marker of an advanced disease status, but also as an active contributor to tumor progression.

## Electronic supplementary material

Below is the link to the electronic supplementary material.

Supplementary Material 1

## Data Availability

The data that support the findings of this study are available from Nagoya University but restrictions apply to the availability of these data, which were used under license for the current study, and so are not publicly available.

## Abbreviations

OvCa: ovarian cancer
PDS: primary debulking surgery
OS: overall survival
PFS: progression-free survival
CT: computed tomography
PS: propensity score
HR: hazard ratio
