# Supplementary material for: Detailed analysis of the histology-specific impact of ascites volume on the outcome of epithelial ovarian cancer: a multi-institutional retrospective cohort study
Source: BMC Cancer. 2024 Nov 29;24:1479. doi: 10.1186/s12885-024-13218-1 (PMC11605864; doi:10.1186/s12885-024-13218-1)
Supplement: Supplementary file 1 — Supplementary Material 1 [file 12885_2024_13218_MOESM1_ESM.docx]

**SUPPLEMENTARY MATERIAL**

**Detailed analysis of the histology-specific impact of ascites volume on the outcome of epithelial ovarian cancer: A multi-institutional retrospective cohort study**

Shohei Iyoshi^1,2#^, Mariko Kimura^3,#^, Masato Yoshihara^1,*^, Atsushi Kunishima^1^, Emiri Miyamoto^1^, Hiroki Fujimoto^1,4^, Kazuhisa Kitami^1,5^, Kazumasa Mogi^1^, Kaname Uno^1^, Sho Tano^1^, Nobuhisa Yoshikawa^1^, Ryo Emoto^6^, Shigeyuki Matsui^6^, Hiroaki Kajiyama^1^

^1^ Department of Obstetrics and Gynecology, Nagoya University Graduate School of Medicine, 65 Tsurumai-cho, Showa-ku, Nagoya 466-8550, Japan

^2^ Institute for Advanced Research, Nagoya University, Furo-cho, Chikusa-ku, Nagoya 464-8601, Japan

^3^ Department of Obstetrics and Gynecology, Okazaki City Hospital, 3-1 Goshoai, Kouryuji-cho, Okazaki 444-8553, Japan

^4^ Discipline of Obstetrics and Gynaecology, Adelaide Medical School, Robinson Research Institute, University of Adelaide, Adelaide, SA, Australia

^5^ Department of Gynecologic Oncology, Aichi Cancer Center, 1-1 Kanokoden, Chikusa-ku, Nagoya 464-8681, Japan

^6^ Department of Biostatistics, Nagoya University Graduate School of Medicine

*Corresponding Author

**Contact information:**

Masato Yoshihara

Department of Obstetrics and Gynecology, Nagoya University Graduate School of Medicine

Showa-ku, Nagoya, Japan

TEL.: +81-52-744-2262

FAX: +81-52-744-2268

E-mail: myoshihara1209@med.nagoya-u.ac.jp

**Contents**

1. Supplementary Tables

2. Supplementary Figure

**Table S1.** The number of cases stratified by histology types and ascites volume


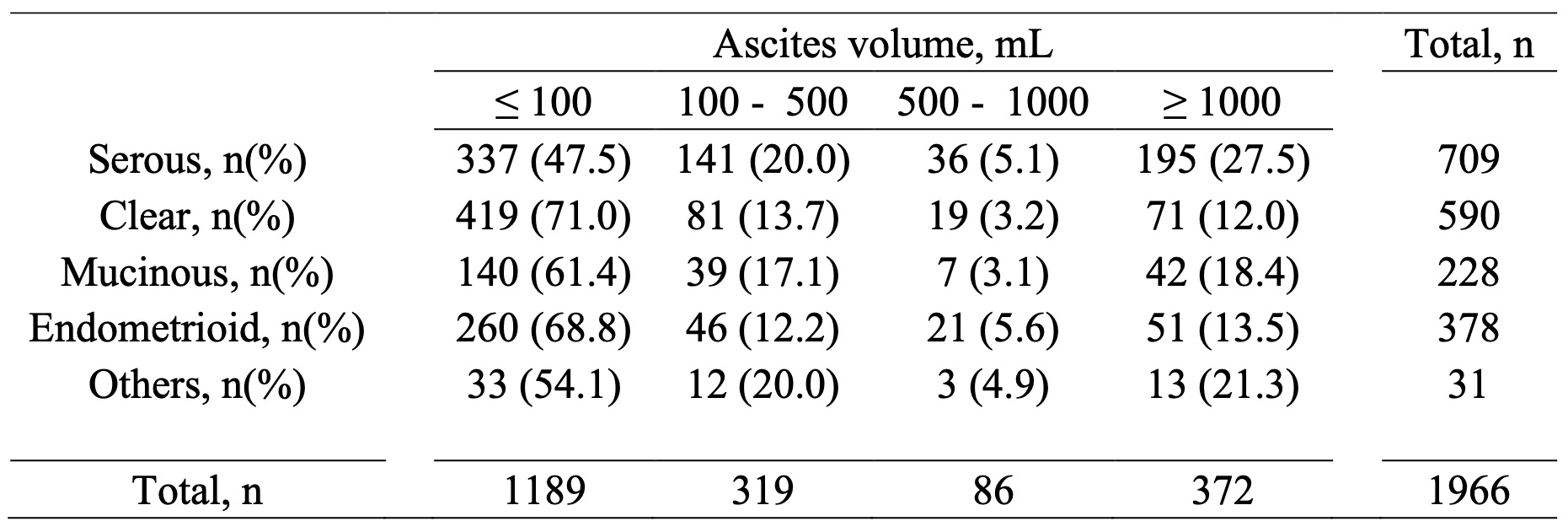

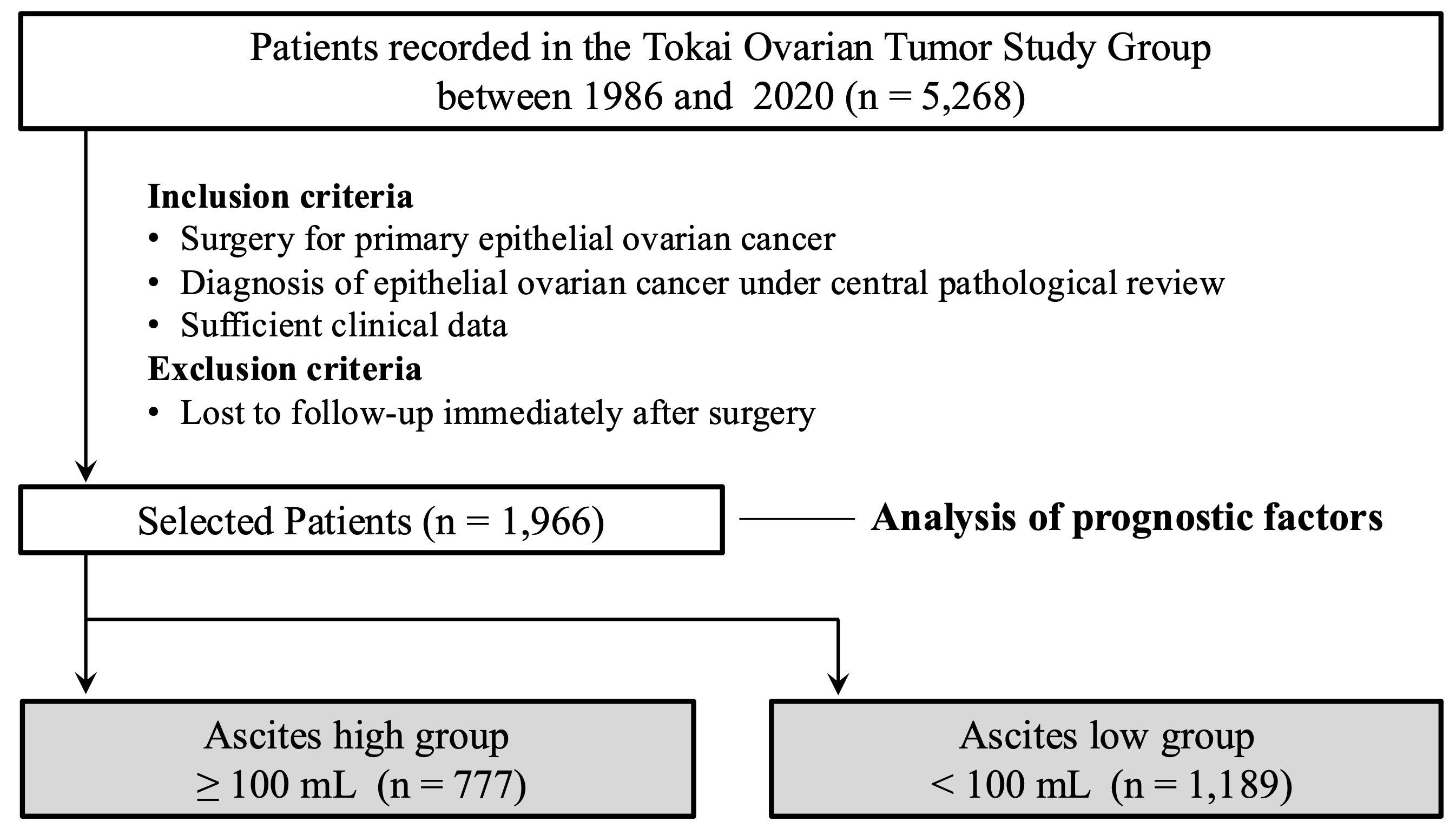


**Figure S1.** Flow chart of the study subjects. Of 5,268 patients with ovarian tumors in the Tokai Ovarian Tumor Study Group between 1986 and 2020, 1,966 cases of epithelial ovarian cancer satisfying inclusion criteria were included in this study.


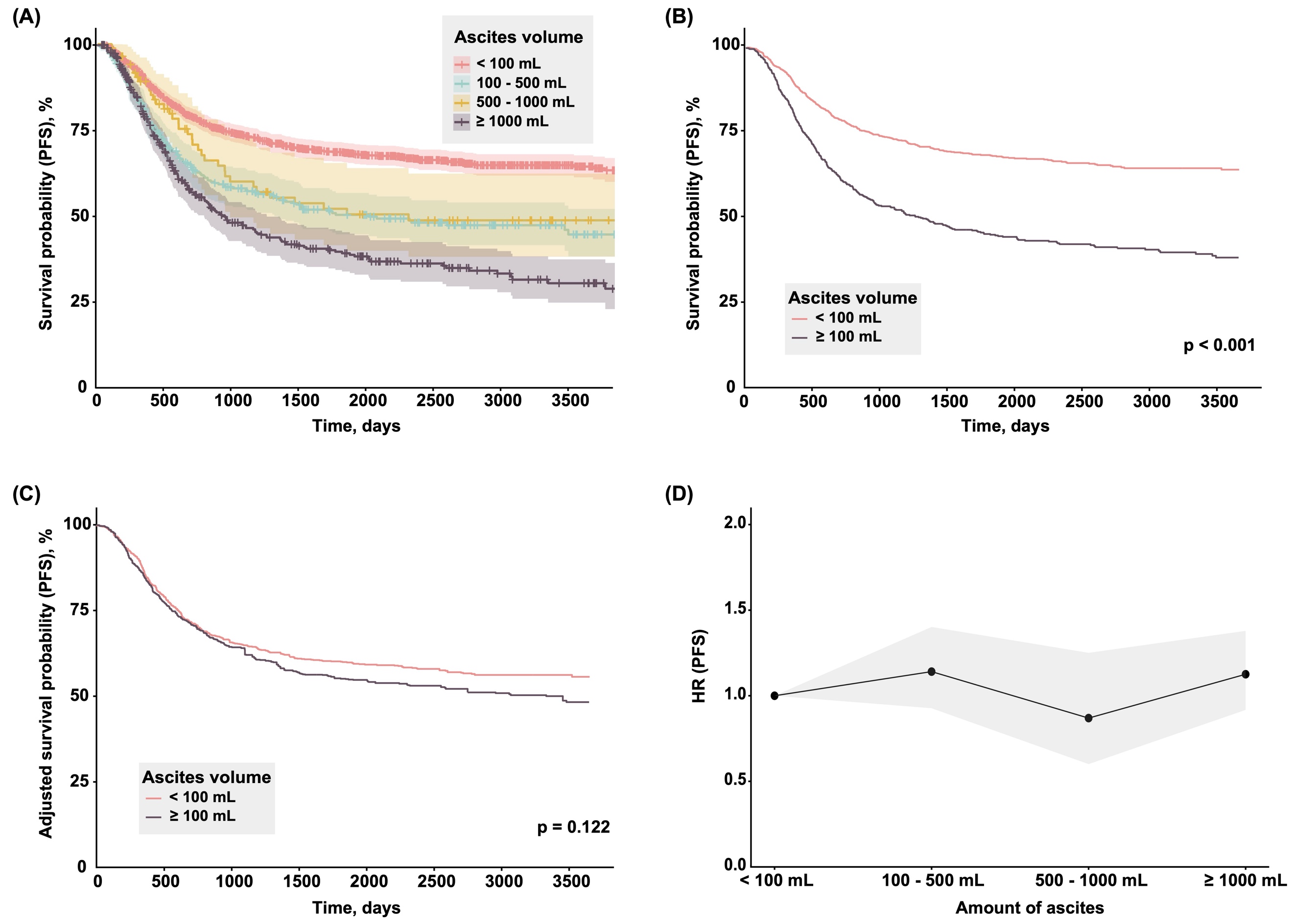


**Figure S2.** (A) Kaplan–Meier curves for PFS stratified by four ascites volume classes (≤100 mL, 100 – 500 mL, 500 – 1000 mL, and ≥1000 mL). (B-C) Kaplan–Meier curves for PFS stratified by two ascites classes (<100 mL and ≥100 mL) with (B) unadjusted and (C) adjusted cohorts. (D) HR values for PFS in each ascites volume class (the HR value of the lowest ascites volume <100 mL was used as a reference) were plotted and 95% confidence intervals were shown as a gray belt.


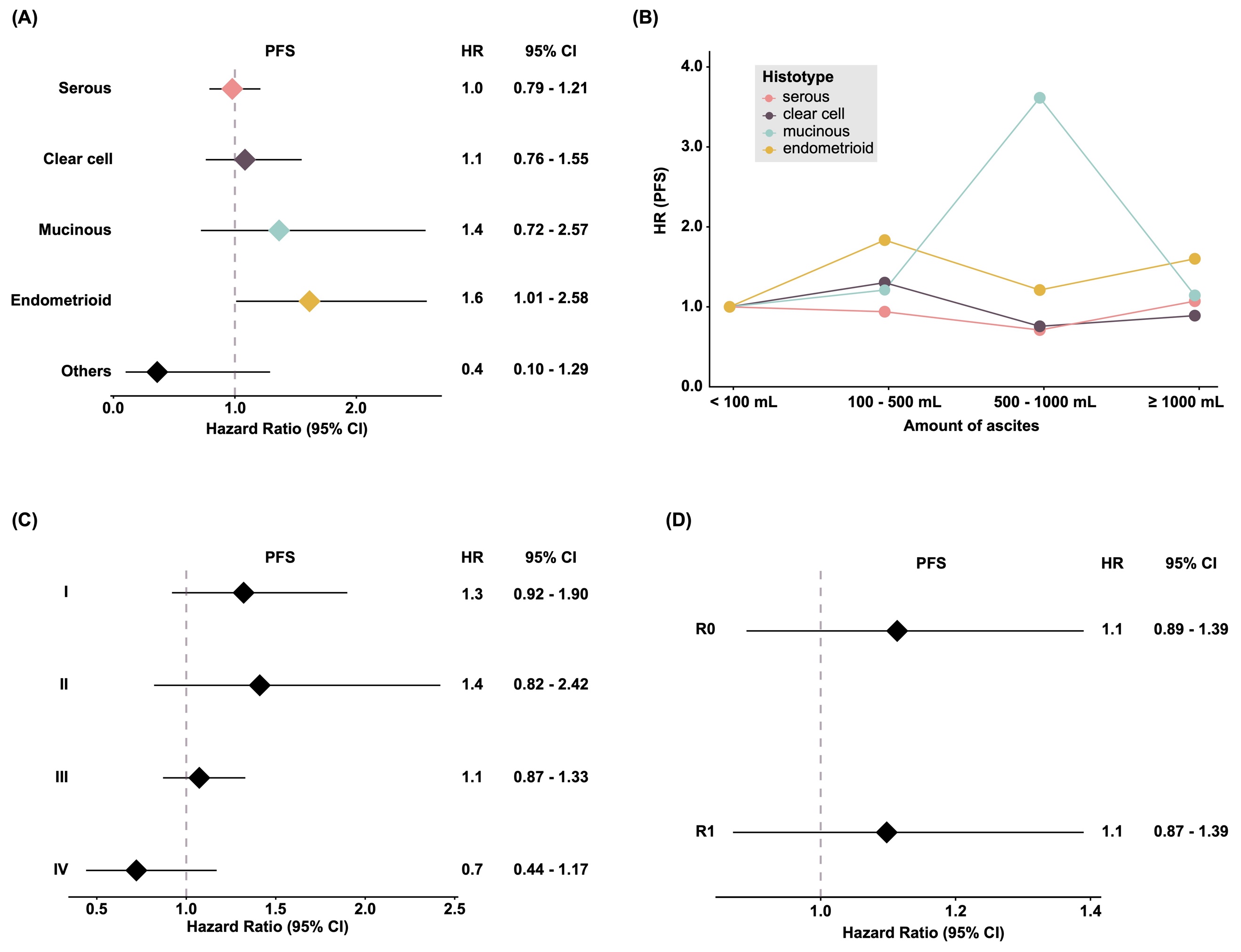


**Figure S3.** (A-D) Results of subgroup analyses for PFS of (A-B) histology, (C) FIGO staging, and (D) R0/R1 surgery groups depicted as HR values.
